# Association of Peritumoral Radiomics With Tumor Biology and Pathologic Response to Preoperative Targeted Therapy for *HER2 (ERBB2)*–Positive Breast Cancer

**DOI:** 10.1001/jamanetworkopen.2019.2561

**Published:** 2019-04-19

**Authors:** Nathaniel Braman, Prateek Prasanna, Jon Whitney, Salendra Singh, Niha Beig, Maryam Etesami, David D. B. Bates, Katherine Gallagher, B. Nicolas Bloch, Manasa Vulchi, Paulette Turk, Kaustav Bera, Jame Abraham, William M. Sikov, George Somlo, Lyndsay N. Harris, Hannah Gilmore, Donna Plecha, Vinay Varadan, Anant Madabhushi

**Affiliations:** 1 Department of Biomedical Engineering, Case Western Reserve University, Cleveland, Ohio; 2 Case Comprehensive Cancer Center, Case Western Reserve University, Cleveland, Ohio; 3 Department of Radiology and Biomedical Imaging, Yale School of Medicine, New Haven, Connecticut; 4 Department of Radiology, Memorial Sloan Kettering Cancer Center, New York, New York; 5 Department of Radiology, Boston Medical Center, Boston, Massachusetts; 6 Department of Radiology, Boston University School of Medicine, Boston, Massachusetts; 7 Department of Hematology and Medical Oncology, The Cleveland Clinic, Cleveland, Ohio; 8 Department of Diagnostic Radiology, The Cleveland Clinic, Cleveland, Ohio; 9 Program in Women’s Oncology, Women and Infants Hospital, Warren Alpert Medical School of Brown University, Providence, Rhode Island; 10 Department of Medical Oncology and Therapeutics Research, City of Hope National Medical Center, Duarte, California; 11 Department of Hematology and Hematopoietic Cell Transplantation, City of Hope National Medical Center, Duarte, California; 12 National Cancer Institute, National Institutes of Health, Bethesda, Maryland; 13 Department of Pathology, University Hospitals Cleveland Medical Center, Cleveland, Ohio; 14 Department of Radiology, University Hospitals Cleveland Medical Center, Cleveland, Ohio; 15 Louis Stokes Cleveland Veterans Administration Medical Center, Cleveland, Ohio

## Abstract

**Importance:**

There has been significant recent interest in understanding the utility of quantitative imaging to delineate breast cancer intrinsic biological factors and therapeutic response. No clinically accepted biomarkers are as yet available for estimation of response to human epidermal growth factor receptor 2 (currently known as *ERBB2*, but referred to as *HER2* in this study)–targeted therapy in breast cancer.

**Objective:**

To determine whether imaging signatures on clinical breast magnetic resonance imaging (MRI) could noninvasively characterize *HER2*-positive tumor biological factors and estimate response to *HER2*-targeted neoadjuvant therapy.

**Design, Setting, and Participants:**

In a retrospective diagnostic study encompassing 209 patients with breast cancer, textural imaging features extracted within the tumor and annular peritumoral tissue regions on MRI were examined as a means to identify increasingly granular breast cancer subgroups relevant to therapeutic approach and response. First, among a cohort of 117 patients who received an MRI prior to neoadjuvant chemotherapy (NAC) at a single institution from April 27, 2012, through September 4, 2015, imaging features that distinguished *HER2*+ tumors from other receptor subtypes were identified. Next, among a cohort of 42 patients with *HER2*+ breast cancers with available MRI and RNaseq data accumulated from a multicenter, preoperative clinical trial (BrUOG 211B), a signature of the response-associated *HER2*-enriched (*HER2*-E) molecular subtype within *HER2*+ tumors (n = 42) was identified. The association of this signature with pathologic complete response was explored in 2 patient cohorts from different institutions, where all patients received *HER2*-targeted NAC (n = 28, n = 50). Finally, the association between significant peritumoral features and lymphocyte distribution was explored in patients within the BrUOG 211B trial who had corresponding biopsy hematoxylin-eosin–stained slide images. Data analysis was conducted from January 15, 2017, to February 14, 2019.

**Main Outcomes and Measures:**

Evaluation of imaging signatures by the area under the receiver operating characteristic curve (AUC) in identifying *HER2*+ molecular subtypes and distinguishing pathologic complete response (ypT0/is) to NAC with *HER2*-targeting.

**Results:**

In the 209 patients included (mean [SD] age, 51.1 [11.7] years), features from the peritumoral regions better discriminated *HER2*-E tumors (maximum AUC, 0.85; 95% CI, 0.79-0.90; 9-12 mm from the tumor) compared with intratumoral features (AUC, 0.76; 95% CI, 0.69-0.84). A classifier combining peritumoral and intratumoral features identified the *HER2*-E subtype (AUC, 0.89; 95% CI, 0.84-0.93) and was significantly associated with response to *HER2*-targeted therapy in both validation cohorts (AUC, 0.80; 95% CI, 0.61-0.98 and AUC, 0.69; 95% CI, 0.53-0.84). Features from the 0- to 3-mm peritumoral region were significantly associated with the density of tumor-infiltrating lymphocytes (*R*^2^ = 0.57; 95% CI, 0.39-0.75; *P* = .002).

**Conclusions and Relevance:**

A combination of peritumoral and intratumoral characteristics appears to identify intrinsic molecular subtypes of *HER2*+ breast cancers from imaging, offering insights into immune response within the peritumoral environment and suggesting potential benefit for treatment guidance.

## Key Points

**Question:**

Can quantitative imaging features extracted from the tumor and tumor environment on breast magnetic resonance imaging characterize tumor biological features relevant to outcome of targeted therapy?

**Findings:**

In this diagnostic study of 209 patients, among *HER2* (*ERBB2*)-positive breast cancers, an intratumoral and peritumoral imaging signature capable of discriminating the response-associated *HER2*-enriched molecular subtype was identified. When evaluated among recipients of *HER2*-targeted therapy, this signature was found to be associated with response to neoadjuvant chemotherapy.

**Meaning:**

Quantitative analysis of the tumor and its surroundings may provide valuable cues into breast cancer biological features and likelihood of response to targeted therapy.

## Teaser

This diagnostic study examines the use of magnetic resonance imaging to identify tumor biological factors associated with targeted therapy response for *HER2*-positive breast cancer.

## Introduction

Human epidermal growth factor receptor 2 (currently known as *ERBB2*, but referred to as *HER2* in this study)–positive breast cancer is morphologically and genetically heterogeneous. Not all patients will fully benefit from *HER2*-targeted treatment, with less than 35% of patients initially responding to therapy with the monoclonal antibody trastuzumab.^1,2^ Molecular profiling via tests such as the *PAM50* gene set can provide insight into treatment response by subcategorizing *HER2*-positive (*HER2*+) tumors into response-associated intrinsic molecular subtypes.^3,4,5,6,7,8^ The *HER2*-enriched (*HER2*-E) subtype, composing 40% to 50% of *HER2*+ breast cancers, is of particular therapeutic interest owing to its elevated rate of response to *HER2*-targeted therapy.^3,9,10^ Although molecular subtyping of *HER2*+ breast cancer is gradually gaining biological significance, no clinically accepted biomarkers are as yet available for prediction of response to anti-*HER2* therapy.^11^ Therefore, there remains a need to develop novel approaches to estimate clinical outcomes of *HER2*-targeted therapy.

In breast cancer, computerized tissue phenotyping on radiographic imaging (or radiomic) features extracted from breast magnetic resonance imaging (MRI) has been shown to be sensitive to many facets of cancer biological factors, such as clinical receptor status,^12,13,14,15,16,17,18,19,20^ genotypic molecular subtype,^21,22,23^ and gene mutation or molecular pathway activation.^24,25,26,27,28,29^ Although some recent approaches have explored direct radiomic estimation of response from pretreatment^30,31^ and interim MRI,^19,32,33^ these approaches often lack well-understood associations with underlying tumor biological factors. While a number of other investigations involving breast radiogenomics (ie, integrating radiomic and genomic data for multiscale tumor characterization) have interrogated biological associations with imaging,^12,13,14,15,16,17,18,19,20,21,22,23,24,25,26,27,28^ relatively little of this work^25,27^ has also placed such findings in the context of clinical outcomes. Thus, the association of radiogenomic signatures with response to targeted therapies remains largely unknown. Similarly, almost all radiogenomic approaches have focused on molecular and genomic correlations with imaging features and not explicitly considered the association of radiomic features with histopathologic attributes. A radiogenomic approach to response assessment, leveraging radiomic signatures of response-associated molecular subtypes with a known morphologic basis, could inform therapeutic approach while still providing biological interpretability.

A growing body of research implicates the tumor microenvironment as a key player in breast cancer development and progression.^34^ Physical and genetic changes within the stroma surrounding a tumor help dictate its ability to grow and spread, evade the body’s immune defenses, and resist therapeutic intervention. Empirical evidence suggests^35^ that the microenvironment might harbor information that enables estimation of treatment response. For instance, an elevated concentration of tumor-infiltrating lymphocytes within the stroma is associated with improved therapeutic outcome in *HER2*+ breast cancer,^36^ and differing immunogenicity between *HER2*+ molecular subtypes of breast cancer has been shown to contribute to their varying treatment outcomes.^4,37,38^ The case for considering the tumor microenvironment is especially strong in the stratification of *HER2*+ by molecular subtype and outcome, as it has recently been shown that *HER2*-E and non–*HER2*-E differ in their interactions with the tumor microenvironment that potentially contribute to therapeutic resistance.^39^

Despite the biological significance of the tumor microenvironment, most breast radiomics approaches have focused on interrogating heterogeneity patterns across the entire tumor^40^ or within intratumoral subregions on breast MRI.^41^ Others have reported success of such approaches within the bulk parenchyma on dynamic contrast enhanced (DCE)–MRI^13,22,23,42,43,44,45^ and other modalities,^43^ indicating the presence of discriminating radiomic information outside of the lesion. In addition, architectural disorder of the surrounding tumor-associated vessel network was recently shown to be associated with treatment response on pretreatment DCE-MRI.^46^ Comparatively few studies,^23,25,30,47^ however, have explored textural measures of heterogeneity within the tumor environment in immediate proximity to the tumor on breast DCE-MRI. This region has been shown to qualitatively differ in appearance on DCE-MRI across intrinsic molecular subtypes of breast cancer^15^ and, thus, radiomic analysis of this region may contribute value to the identification of the *HER2*-E subtype. In previous work, supplementing analysis of the tumor with peritumoral radiomics—textural measurements within the tissue surrounding the tumor—enabled the estimation of treatment response on pretreatment DCE-MRI.^30^ One hypothesis for the estimative capability of peritumoral radiomics is that these features might detect the magnitude of pretreatment immune response and spatial architecture of lymphocytes within the tumor environment.^30^

In this study, we evaluated response-associated subtypes of *HER2*+ breast cancer by interrogating the tumor and peritumoral environment on imaging. We then examined a possible association between radiogenomic signature of *HER2*-E and response to *HER2*-targeted neoadjuvant chemotherapy (NAC) in 2 independent validation cohorts. We also explored the underlying biological basis of this distinctive radiomic signature through a quantitative comparison with pathologic immune response. Our approach represents several possible contributions to the area of breast radiogenomics: (1) radiogenomic subtyping of *HER2*+ breast cancer using both intratumoral and peritumoral textural patterns, (2) applying radiogenomic subtyping to the assessment of response to a specific targeted therapy, and (3) substantiating radiogenomic signatures through morphologic association with corresponding biopsy samples.

## Methods

### Data Sets and Experiments

**Figure 1.**
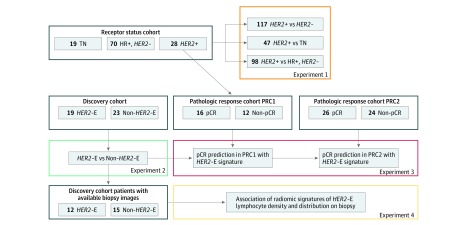
Experimental Design eFigure 2 in the Supplement depicts the process of developing imaging signatures associated with receptor status (experiment 1) and *HER2*+ molecular subtype (experiment 2). *HER2*-E indicates *HER2*-enriched; HR, hormone receptor; pCR, pathologic complete response; PRC1, pathologic response cohort 1; PRC2, pathologic response cohort 2; and TN, triple negative.

The flowchart in Figure 1 depicts an overview of the data sets used in this study and the various experiments performed. Clinical and scan information for each cohort is either described in a previous publication^30^ or included in Table 1. The analysis included 209 patients (mean [SD] age, 51.1 [11.7] years). This Health Insurance Portability and Accountability Act of 1996 regulations–compliant study was approved by the institutional review board at the University Hospitals Cleveland Medical Center, Cleveland, Ohio, and the need for informed consent was waived; a correlative study was also conducted after review and approval by the University Hospitals Cleveland Medical Center Institutional Review Board. This study followed the Standards for Reporting of Diagnostic Accuracy (STARD) reporting guideline. The study was conducted from April 27, 2012, through September 4, 2015, and data analysis was performed from January 15, 2017, to February 14, 2019.

**Table 1.** Clinical Information for the BrUOG 211B/TCIA Molecular Subtype Discovery Cohort and PRC1 and PRC2

Variable	Discovery		PRC1		PRC2		*P* Value
	*HER2*-E	Non–*HER2*-E	pCR	Non-pCR	pCR	Non-pCR	
No. of patients	19	23	16	12	26	24	
Age, mean (SD), y^a^	50.9 (7.7)	51.7 (9.8)	47.9 (13.4)	47.4 (11.7)	49.7 (11.2)	50.7 (13.7)	.41
Receptor status, No.							
ER+	4	21	8	8	13	22	.43^b^
PR+	2	17	7	8	9	16	.78^b^
Stage, No.							
I	1	3	1	3	3	2	.55^b^
II	12	12	9	7	16	18	
III	5	8	6	1	7	4	
IV	0	0	0	1	0	0	
NA	1	0	0	0	0	0	
Scanner strength, No.							
1.5 T	18	19	14	9	26	24	NA
3 T	1	4	2	3	0	0	NA
Scanner make/model, No.^c^							
Scanner 1	0	2	8	6	9	4	NA
Scanner 2	1	4	5	2	17	18	NA
Scanner 3	10	8	0	1	0	2	NA
Scanner 4	8	5	2	2	0	0	NA
Scanner 5	0	4	1	1	0	0	NA
Treatment regimen, No.							
DCT	NA	NA	2	3	0	0	NA
DCTP	NA	NA	14	9	26	24	NA
Surgical intervention, No.							
Breast-conserving surgery	NA	NA	5	6	11	6	NA
Mastectomy	NA	NA	11	6	15	18	NA
Biopsy sample available, No.	12	15	NA	NA	NA	NA	NA
Contained peripheral tissue	5	8	NA	NA	NA	NA	NA

Abbreviations: DCT, docetaxel, carboplatin, and trastuzumab; DCTP, docetaxel, carboplatin, trastuzumab, and pertuzumab; ER+, estrogen receptor–positive; *HER2*-E, *HER2*-enriched; NA, not applicable; pCR, pathologic complete response; PRC1, pathologic response cohort 1; PRC2, pathologic response cohort 2; PR+, progesterone receptor–positive.

^a^ No significant difference in mean of PRC1 and PRC2 compared with the discovery cohort by unpaired, 2-sided *t* test.

^b^ No significant difference in categorical distribution of PRC1 and PRC2 compared with the discovery cohort by Pearson χ^2^ test.

^c^ Scanner models differ between cohorts and are listed within the same rows for simplicity. Discovery cohort*:* scanner 1, Siemens Avanto; scanner 2, Siemens Verio; scanner 3, Siemens Symphony or SymphonyTim; scanner 4, General Electric (GE) Medical Systems Signa Excite; scanner 5, GE Medical Systems Signa Hdx or Hdxt. PRC1: scanner 1, Siemens Avanto; scanner 2, Siemens Espree; scanner 3, Siemens Verio; scanner 4, Philips Medical Systems Ingenuity; scanner 5, Philips Medical Systems Intera. PRC2: scanner 1, Siemens Avanto; scanner 2, Siemens Espree; scanner 3, Siemens Aera.

#### Distinguishing Receptor Subtypes

A previously described cohort of 117 patients^30^ who received neoadjuvant treatment at University Hospitals Cleveland Medical Center was used to first assess the ability of peritumoral radiomics to differentiate *HER2*+ from breast cancers of other receptor statuses. This cohort contained 28 *HER2*+ and 89 *HER2-*negative (*HER2*−) breast cancers (70 hormone receptor–positive [HR+], and 19 triple negative [TN]) receptor status. Several signatures were developed and evaluated in cross-validation within this data set to distinguish *HER2*+ from (1) HR+, *HER2*−; (2) TN; and (3) all *HER2*− tumors.

#### Molecular Subtyping of *HER2*+ 

A retrospective, multi-institutional data set of 42 patients with *HER2*+ breast cancer with pre-NAC DCE-MRI scan findings (eMethods in the Supplement) and gene expression data available formed the molecular subtype discovery cohort. Data on 35 patients were obtained from the BrUOG 211B multicenter, preoperative clinical trial^48^ accrued between June 5, 2008, and August 13, 2012, at Brown University Oncology Research Group participating hospitals, Providence, Rhode Island, Yale Cancer Center, New Haven, Connecticut, and City of Hope Comprehensive Cancer Center, Duarte, California, with written informed consent. Seven patients from the Cancer Genome Atlas–Breast Cancer (TCGA-BRCA) project with imaging results available through the Cancer Imaging Archive (TCIA)^49,50^ were also included. The patient selection flowchart for this cohort is included in eFigure 1 in the Supplement and the distribution of clinical variables in the discovery cohort is compared with the original study populations in eTable 1 in the Supplement.

*HER2 *positivity was confirmed by either overexpression by immunohistochemistry stain (3+) or a fluorescent in situ hybridization ratio for *HER*/CEP17 greater than 2.0. Intrinsic subtyping was described in greater detail previously.^4^ Briefly, unsupervised clustering of PAM50 gene expression values, quantified by microarray or targeted RNASeq of biopsy samples, was performed, and clusters were assigned to luminal, basal, and *HER2*-E subgroups based on estrogen receptor (ER) and/or progesterone receptor (PR) IHC values and relative expression of the proliferation-associated genes within the PAM50 gene list.^4,51^ Nineteen patients were assigned the *HER2*-E subtype, whereas the remaining 23 were assigned non–*HER2*-E subtypes (19 *HER2*-luminal, 4 *HER2*-basal). Imaging signatures capable of distinguishing *HER2*-E from *HER2*+ were developed and evaluated in this cohort via cross-validation.

#### Association With *HER2*-Targeted Therapy Response

We further evaluated our *HER2*-E signature by assessing its association with pathologic complete response (pCR) to *HER2*-targeted NAC in 2 retrospective pathologic response cohorts. The first cohort was pathologic response cohort 1 (PRC1). The 28 *HER2*+ University Hospitals patients described previously^30^ were additionally used for initial evaluation of response association. Sixteen achieved pCR on surgical specimen (ypT0/is), and 12 retained the presence of residual disease following NAC (non-pCR). Twenty-three patients in PRC1 received a combination of docetaxel, carboplatin, trastuzumab, and pertuzumab (DCTP) and 5 received only docetaxel, carboplatin, and trastuzumab (DCT). The second cohort was pathologic response cohort 2 (PRC2). Fifty *HER2*+ patients (26 pCR, 24 non-pCR by ypT0/is) who received DCE-MRI scans before *HER2*-targeted NAC at the Cleveland Clinic were used to further validate the association of the radiogenomic signature of *HER2*-E with response. All patients in PRC2 were scanned using 1.5-T Siemens scanners and received DCTP.

#### Association With Lymphocyte Distribution 

Twenty-seven patients from the BrUOG 211B trial molecular subtyping cohort had hematoxylin-eosin–stained slides and slide images of pretreatment biopsy samples also available. A post hoc radiology-pathology correlation experiment was performed to assess associations between radiomic signatures within the peritumoral tissue and pretreatment immune response as measured by tumor-infiltrating lymphocyte (TIL) density. For the subset of biopsy samples containing sufficient peripheral nontumor tissue for analysis (n = 13), additional correlative analysis was performed with peritumoral lymphocytic density.

### Lesion Segmentation and Feature Extraction

Images were scaled within a standardized intensity range based on maximum and minimum intensity values. Multiple readers (M.E., D.D.B.B., K.G., B.N.B., P.T., K.B., and D.P.) provided annotations on 3 adjacent slices of DCE-MRI scans working in partial consensus, which were then used to derive 5 annular rings of 3 mm each (excluding skin, air, or pectoralis muscle) out to a maximum distance of 15 mm, consistent with previous studies analyzing the tumor environment.^25,52,53^ Ninety-nine texture descriptors were extracted from each region, composing the following 4 feature groups (eMethods and eFigure 2 in the Supplement provide further details): (1) 25 Laws descriptors,^54^ capturing combinations of 5 irregular enhancement patterns, such as level, edges, spots, waves, or ripples; (2) 48 Gabor descriptors,^55^ capturing wavelike patterns of intensity variations across 6 different spatial scales (2, 4, 8, 16, 32, and 64 pixels [px]) at 8 directional orientations (0°, 22.5°, 45°, 67.5°, 90°, 112.5°, 135°, 157.5°); (3) 13 gray level co-occurrence matrix (GLCM) descriptors,^56^ capturing the heterogeneity of adjacent intensity values within local pixel neighborhoods; and (4) 13 co-occurrence of local anisotropy gradients (CoLlAGe) descriptors,^57^ capturing structural disorder by applying GLCM heterogeneity metrics to directional intensity patterns.

First-order statistics (mean, median, SD, skewness, kurtosis) for each descriptor were computed within the tumor and each peritumoral annulus, yielding 495 statistical features per region. Features were normalized based on mean and SD within the training cohort.

### Feature Selection

Feature selection was performed within each region and across all regions. Owing to the high dimensionality of our feature pool, highly correlated features from each class were removed before feature selection. Groups of correlated features (Pearson linear correlation coefficient ≥0.6) were identified and all but the single most significant feature determined by unpaired, 2-sided *t* test were eliminated. For the 4 classes of descriptors, a total of 6 to 9 (Laws), 8 to 11 (GLCM), 55 to 65 (Gabor), and 10 to 15 (COLlAGe) features within individual regions and 29 (Laws), 41 (GLCM), 207 (Gabor), and 70 (COLlAGe) features across all regions combined remained. Imaging signatures were limited to 5 features to reduce the risk of overfitting, and the top features were identified as those most frequently selected across 500 iterations of feature selection within the pool of uncorrelated features in a 3-fold, cross-validation setting. Features were selected one at a time by Bhattacharyya distance,^58,59^ weighted by correlation with previously selected features to further reduce redundancy and overfitting. Feature extraction and selection pipeline are depicted in eFigure 2 in the Supplement.

### Statistical Analysis

A diagonal linear discriminant analysis classifier^60^ incorporating each set of top features was trained and assessed through 100 iterations of 3-fold cross-validation within training cohorts. A final *HER2*-E classifier was trained and locked down using the entire discovery cohort, then evaluated for association with response in the retrospective validation data sets (PRC1, PRC2). Significance of the area under the curve (AUC) was determined via permutation testing with random sampling^61,62^ (eMethods in the Supplement). The significance of AUC improvement when incorporating both intratumoral and peritumoral features was assessed by paired, 1-sided Delong test for correlated area under the receiver operating characteristic (ROC) curves.^63^ Operating points on the ROC curve for calculation of sensitivity and specificity were chosen according to the Youden Index.^64^

A previously developed automated nuclei and lymphocyte detection model^65^ was adapted to detect lymphocytes on hematoxylin-eosin–stained slides of pretreatment biopsy samples for patients from the BrUOG 211B trial (model training and validation described in eMethods in the Supplement). Pathologic immune response was quantified as the number of lymphocytes per unit area separately within and beyond pathologist-annotated tumor boundaries. Multivariable linear regression models of lymphocytic density within the tumor and surrounding tissue were developed from the top 5 features for each imaging region. Significance of the *R*^2^ statistic was determined by *F* test of error variance with Benjamini-Hochberg multiple comparison correction.^66,67^

## Results

### Distinguishing Receptor Status

To first establish a basis for peritumoral radiomics in the context of characterizing *HER2*+ biological features, we investigated their capability to distinguish *HER2*+ breast cancer from other clinical receptor status groups. The addition of peritumoral radiomic features improved the ability to distinguish *HER2*+ vs HR+ (AUC, 0.71; 95% CI, 0.67-0.75; *P* < .001; n = 98), TN (AUC, 0.80; 95% CI, 0.76-0.84; *P* < .001; n = 47), and all other subtypes (AUC, 0.65; 0.59-0.71; *P* = .006) compared with intratumoral features alone. The AUC and top feature sets for all comparisons with and without peritumoral features are listed in Table 2.

**Table 2.** Features and Performance for Intratumoral Only and Combined Intratumoral and Peritumoral Region Classifiers in Distinguishing *HER2*+ From Other Receptor Subtypes and Stratifying *HER2*+ by Molecular Subtype

Region	Feature				Signature Performance	
	Group	Descriptor	Statistic	*P* Value	AUC (95% CI)	*P* Value
***HER2*+ vs HR-Positive, *HER2−***						
Intratumoral						
	Gabor	Width, 6 px; orientation, 67.5°	Kurtosis	.01	0.69 (0.65-0.73)	<.001
	GLCM	Energy	Kurtosis	.10		
	Gabor	Width, 8 px; orientation, 67.5°	Kurtosis	.08		
	Laws	Spot-edge	Median	.007		
	CoLlAGe	Sum average	Skewness	.003		
Intratumoral and peritumoral					0.71 (0.67-0.75)	<.001
Tumor	Gabor	Width, 16 px; orientation, 67.5°	Kurtosis	.01		
Tumor	GLCM	Energy	Kurtosis	.10		
9-12 mm	Gabor	Width, 32 px; orientation, 112.5°	Kurtosis	.02		
Tumor	Laws	Spot-edge	Median	.007		
Tumor	CoLlAGe	Sum average	Skewness	.003		
***HER2*+ vs TN**						
Intratumoral					0.73 (0.67-0.79)	.002
	Laws	Edge-level	Median	.05		
	Gabor	Width, 8 px; orientation, 45°	Kurtosis	.22		
	Laws	Ripple-ripple	Kurtosis	.06		
	Gabor	Width, 2 px; orientation, 0°	Kurtosis	.16		
	GLCM	Energy	Skewness	.12		
Intratumoral and peritumoral					0.80 (0.76-0.84)	<.001
9-12 mm	Gabor	Width, 4 px; orientation, 90°	SD	<.001		
Tumor	Gabor	Width, 8 px; orientation, 45°	Kurtosis	.22		
Tumor	Laws	Edge-level	Median	.05		
9-12 mm	Gabor	Width, 4 px; orientation, 67.5°	Mean	<.001		
9-12 mm	GLCM	Sum variance	Kurtosis	.49		
***HER2*+ vs All**						
Intratumoral						
	Gabor	Width, 6 px; orientation,67.5°	Kurtosis	.01	0.65 (0.59-0.71)	0.006
	GLCM	Energy	Kurtosis	.05		
	Gabor	Width, 8 px; orientation, 67.5°	Kurtosis	.05		
	Laws	Spot-edge	Median	.02		
	Gabor	Width, 8 px; orientation, 45°	Kurtosis	.05		
Intratumoral and peritumoral						
Tumor	Gabor	Width, 16 px; orientation, 67.5°	Kurtosis	.01	0.71 (0.63-0.79)	<.001
Tumor	GLCM	Energy	Kurtosis	.05		
6-9 mm	Laws	Ripple-ripple	Kurtosis	.05		
Tumor	Laws	Spot-edge	Median	.02		
0-3 mm	GLCM	Info2	Kurtosis	.05		
***HER2*-E vs Non–*HER2*-E**						
Intratumoral						
	Gabor	Width, 4 px; orientation, 135°	Kurtosis	.02	0.76 (0.69-0.84)	<.001
	Laws	Ripple-ripple	Kurtosis	.02		
	Gabor	Width, 16 px; orientation, 112.5°	Kurtosis	.05		
	Gabor	Width, 16 px; orientation, 45°	Kurtosis	.43		
	CoLlAGe	Energy	Kurtosis	.03		
Intratumoral and peritumoral						
Tumor	Laws	Ripple-Ripple	Kurtosis	.02	0.89 (0.84-0.93)	<.001
Tumor	Gabor	Width, 16 px; orientation, 112.5°	Kurtosis	.05		
6-9 mm	CoLlAGe	Energy	Kurtosis	.04		
Tumor	Gabor	Width, 4 px; orientation, 135°	Kurtosis	.02		
9-12 mm	CoLlAGe	Inertia	Median	.002		

Abbreviations: AUC, area under the receiver operating characteristic curve; CoLlAGe, co-occurrence of local anisotropic gradient orientation features; GLCM, Gray level co-occurrence matrix features; *HER2*-E, *HER2*-enriched; HR, hormone receptor; px, pixels; TN, triple-negative.

### Molecular Subtyping of *HER2*+ 

**Figure 2.**
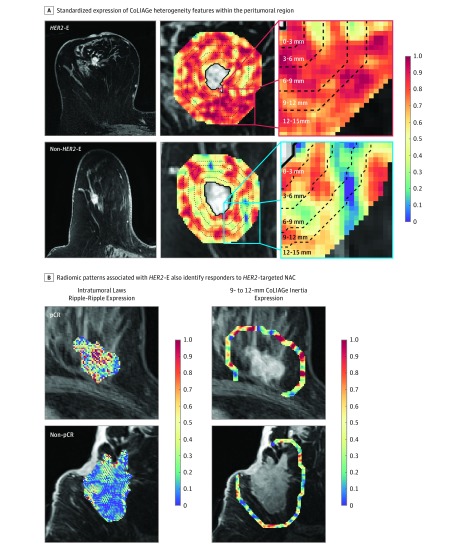
Peritumoral Signature of *HER2*-Enriched (*HER2*-E) Identifies Responders to *HER2*-Targeted Therapy A, Co-occurrence of local anisotropy gradients (CoLlAGe) feature expression maps visualize the elevated disorder of local intensity gradient orientations within the peritumoral region of *HER2*-E relative to non–*HER2*-E breast cancers. B, Imaging signature of *HER2*-E is also associated with pathologic complete response (pCR) to anti-*HER2* therapy, with rippled enhancement patterns detected intratumorally by Laws feature and elevated local peritumoral heterogeneity captured by CoLlAGe features 9 to 12 mm from the tumor characterizing both features. NAC indicates neoadjuvant chemotherapy. Radiomic feature values are unitless, thus the scale depicts relative expression values of radiomic features, standardized between 0 and 1.0 based on the range of their distribution. The blue color at 0 depicts the minimum observed feature value; the red color at 1.0 depicts the maximum observed feature value.

A signature of intratumoral features stratified the response-associated *HER2*-E subtype from other nonenriched *HER2*+ tumors with a mean AUC of 0.76 (95% CI, 0.69-0.84). Within all individual regions beyond the tumor examined, peritumoral features outperformed intratumoral features (maximum cross-validated AUC, 0.85; 95% CI, 0.79-0.90, within the 9- to 12-mm region). Within and near the tumor, Gabor features were most frequently selected. With greater peritumoral radius, CoLlAGe features quantifying the elevated disorder of local intensity gradient orientations in *HER2*-E became more predominant (Figure 2A), such as in the 6- to 9-mm region where CoLlAGe comprised all but 1 top feature (eTable 2 in the Supplement). Full feature sets and AUCs for each peritumoral region are included in eTable 2 in the Supplement. Nonparametric feature elimination methods were also assessed and found to select overlapping feature sets and yield similar performance (AUC, 0.84; 95% CI, 0.80-0.88 with pruning by Spearman correlation and 0.87; 95% CI, 0.81-0.93 with pruning by elastic net regularization) (eTable 3 in the Supplement).

A combined intratumoral and peritumoral feature set identified across features from the intratumoral and all peritumoral regions (Table 2) best stratified *HER2*+ molecular subtypes. This feature set included 3 intratumoral, filter-based features (2 Gabor and 1 Laws) and 2 peritumoral CoLlAGe texture entropy features from the 6- to 9-mm and 9- to 12-mm regions. *HER2*-E was identified with cross-validated AUC (0.89; 95% CI, 0.84-0.93), which was a significant improvement (*P* = .04) over intratumoral features only. Mean classification performance between models with and without peritumoral models were further compared via risk stratification (eTable 4 in the Supplement). Output of the combined intratumoral and peritumoral model was found to offer significant independent value (*P* = .007) when combined in a multivariate setting with clinical variables, such as age, ER status, PR status, and stage (eTable 5 in the Supplement).

### Association With *HER2*-Targeted Therapy Response

In 2 pathologic response cohorts, our *HER2*-E radiomic signature was found to be associated with pCR to preoperative anti-*HER2* therapy, consistent with the molecular subtype elevated rate of response in this context.^4^ In PRC1, the combined peritumoral and intratumoral feature set produced the only classifier significantly associated (*P* = .003) with response on pretreatment imaging, yielding an AUC of 0.80 (95% CI, 0.61-0.98), with accuracy of 79%, sensitivity of 94%, and specificity of 58% at the operating point. This model was again found to offer independent value in a multivariate comparison with clinical variables, this time in the context of pCR estimation in PRC1 (eTable 6 in the Supplement). Meanwhile, intratumoral features alone failed to significantly distinguish pCR (AUC, 0.66; 95% CI, 0.43-0.88; *P* = .08) with poorer classification results (accuracy, 68%; sensitivity, 44%; specificity, 100%), along with individual peritumoral regions (eTable 2 in the Supplement). Figure 2B depicts representative heatmaps corresponding to top intratumoral (Laws ripple-ripple) and peritumoral (CoLlAGe inertia) features. As with *HER2*-E, expression of these features was elevated in patients who achieve pCR compared with other *HER2*+ breast cancers. Breast Imaging Reporting and Data System assessment of background parenchymal enhancement and fibroglandular tissue volume did not differ significantly between response groups (eTable 7 in the Supplement).

The combined peritumoral and intratumoral classifier was further evaluated in its ability to predict response in PRC2. The classifier again significantly distinguished between pCR and non-pCR, with an AUC of 0.69 (95% CI, 0.53-0.84; *P* = .02). Accuracy, sensitivity, and specificity were 68%, 62%, and 75%, respectively. ROC curves for the combined feature model within PRC1 and PRC2 are depicted in eFigure 3 in the Supplement.

### Association With Lymphocyte Density

**Figure 3.**
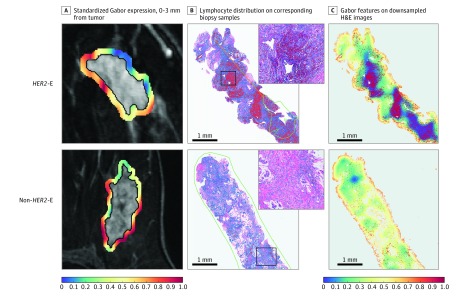
Molecular Subtype Signatures Within the Peritumoral Region Associated With Lymphocyte Density and Distribution on Biopsy A, Kurtosis of Gabor features 0 to 3 mm from the tumor on magnetic resonance imaging, associated with *HER2-*enriched (*HER2*-E) status was additionally associated with B, lymphocyte density within and 0-3 mm beyond the tumor on corresponding biopsy samples. Red and blue dots indicate lymphocytes and other nuclei, respectively. Green lines denote pathologist-annotated tumor boundaries (hematoxylin-eosin; inset original magnification ×100). C, When hematoxylin-eosin–stained images are down-sampled to approximate the imaging scale (original magnification ×100), midfrequency Gabor features computed on example hematoxylin-eosin images at that magnification possess spatial association with lymphocyte density. Radiomic feature values are unitless, thus the scale depicts relative expression values of radiomic features, standardized between 0 and 1.0 based on the range of their distribution. The blue color at 0 depicts the minimum observed feature value; the red color at 1.0 depicts the maximum observed feature value.

Qualitative associations have been observed between peritumoral texture and TIL presence at the tumor margins on biopsy and posited elevated immune response as a potential biological underpinning of predictive radiomic signatures in the surrounding tumor environment.^30^ The lymphocyte detection model successfully identified TILs and peripheral lymphocytes on hematoxylin-eosin–stained biopsy slide images (eFigure 4 in the Supplement). The top 5 features within the peritumoral region closest to the tumor (0-3 mm) was the only region significantly associated (eFigure 5A in the Supplement) with TIL density following Benjamini-Hochberg correction for multiple comparisons (*R*^2^ = 0.57; 95% CI, 0.39-0.75; *P* = .002). The DCE-MRI feature expression maps for one of these features, Gabor (width, 16 px; orientation, 67.5°), are shown alongside detected TILs on corresponding biopsy samples and lymphocytes in Figure 3A and B, respectively. Gabor features computed on hematoxylin-eosin–stained slides (down-sampled to ×1 original magnification to approximate the radiologic scale) show a spatial association between reduced expression and dense lymphocyte distribution (Figure 3C)—a pattern mirroring the correlation first observed between DCE-MRI and histologic characteristics. Peripheral lymphocytic density was observed to be more strongly correlated with radiomic features the greater the distance from the tumor (eFigure 5B in the Supplement). However, none of these correlations was significant, potentially owing to the limited number of biopsy samples with sufficient peripheral tissue for analysis (n = 13).

## Discussion

Although the advent of *HER2*-targeted therapy has improved prognosis for *HER2*+ breast cancer,^68^ a large percentage of *HER2*+ tumors will nonetheless fail to achieve optimal preoperative response to a combination of chemotherapy and anti-*HER2* therapy.^1,2^ In this study, the findings suggest that DCE-MRI peritumoral radiomics may enable noninvasive intrinsic subtyping of *HER2*+ breast cancer into response-associated subgroups. Features from all peritumoral regions better individually identified *HER2*-E breast cancers than analysis of the tumor itself. A combined peritumoral and intratumoral signature of *HER2*-E on pretreatment MRI was found to be significantly associated with pCR, consistent with the *HER2*-E subgroup’s superior response to *HER2*-targeted therapy compared with other *HER2*+ breast cancers.^4^ This association was supported in 2 independent validation cohorts from different institutions: one with high heterogeneity (PRC1: mixed magnetic strengths and scanner manufacturers, multiple treatment regimens, variability in voxel size) and the other with homogeneous treatment and MRI acquisition protocols.

A growing body of work^23,25,30,47^ suggests that the adjacent peritumoral tissue on MRI can provide unique insight into breast cancer biological features and outcomes. The *HER2*+ tumor environment is an especially attractive target for radiogenomic subtyping, as it contains a wide range of prognostic factors that vary between its molecular subtypes.^34^ Our findings provide new insight into *HER2*+ tumor biological characteristics and its radiographic phenotype, as the superior discriminability of peritumoral radiomic features appears to suggest discriminable differences of the tumor environment between the intrinsic molecular subtypes of *HER2*+ breast cancer. *HER2*-E was best characterized by a combination of local disorder, particularly within the peritumoral environment, and macroscale homogeneity near the tumor. Elevated expression of CoLlAGe features capturing chaotic orientation of local intensity gradients within the outer peritumoral regions was an important component of the *HER2*-E radiomic signature. *HER2*-E was also characterized by homogeneity at the macroscale both within and near the tumor, as detected by midwavelength Gabor features. Peritumoral radiomics also improved the capability to distinguish *HER2*+ from other breast cancers, such as TN. Our findings are consistent with those of Li et al^21^ and Waugh et al,^19^ who observed elevated intratumoral texture entropy among *HER2*-E and *HER2*+ HR− tumors, respectively.

We hypothesize that an elevated immune response and spatial arrangement of lymphocytes surrounding *HER2*-E tumors might contribute to this unique peritumoral signature. A robust immune response could, through mechanisms such as immune infiltration and inflammation, result in the local heterogeneity within the tumor environment captured by CoLlAGe and Laws features. Simultaneously, at the scale captured by Gabor features, that same immune response might appear to be more smoothly textured than tissue with sparse lymphocyte infiltration intermixed with healthy, tumor, and fibrotic tissue. We observed a significant correlation between peritumoral radiomic features immediately outside the tumor and lymphocytic density on pretreatment biopsy samples. We noted in particular that reduced expression of middle-frequency Gabor features within this region on DCE-MRI was associated with high lymphocytic density, which was a trend further evidenced by spatial colocalization of Gabor features and lymphocytic density on example down-sampled hematoxylin-eosin–stained images. These findings may indicate a robust immune response detectable at the imaging scale through peritumoral analysis, but they will require further confirmation.

Such imaging associations with immune response have been corroborated in previous studies. Wu et al^25^ reported an association between peritumoral heterogeneity and a gene signature partially associated with immune cell recruitment and inflammation. Others have reported high lymphocytic infiltration to be associated with texture entropy features,^69^ qualitative tumor enhancement profile and margin appearance in TN tumors,^70^ and background parenchymal enhancement within 20 mm from the tumor.^71^ Associations between intratumoral^72^ and peritumoral^73^ textural heterogeneity features with immune response at the molecular and morphometric scale have also been reported in the context of lung computed tomography. Recently, Chen et al^74^ found that incorporating peritumoral radiomic analysis of hepatocellular cancer on contrast-enhanced MRI significantly improved the capability to estimate the immunoscore of TIL density and arrangement compared with a model containing only intratumoral features. Although our study explored peritumoral radiomic associations with pathologic immune response, other biological factors may also contribute to the unique DCE-MRI peritumoral signature of *HER2*-E breast cancer, such as microvessel density,^47^ proliferation,^26^ and necrosis.^25^

### Strengths and Limitations

This work contributes to the area of breast radiomics and radiogenomics in the following ways. First, to our knowledge, this study is the first to explore the role of the peritumoral environment in radiogenomic subtyping from breast cancer MRI and holds important implications regarding the biological characteristics and differential response of *HER2*+ subtypes. Second, we simultaneously addressed both radiogenomic subtyping and response estimation by applying an imaging signature of a response-associated genotype to directly identify therapeutic response. By using an approach that combines estimative radiomics and radiogenomics, we hope to achieve both the clinical relevance of the former with the biological interpretability of the latter. Third, we explored the morphologic basis of our radiogenomic features through correlation with patterns of immune infiltration on histologic findings. Thus, this work represents a possible novel confluence of radiomics, genomics, and digital pathologic features for the purpose of biologically validated response estimation.

Our study has limitations. First, we were able to obtain data on only 42 patients with *HER2*+ tumors with both genomic and imaging information to form our discovery cohort. We performed independent testing in the context of response estimation and correlation with histomorphologic immune response to further substantiate our radiogenomic *HER2*-E signature; however, validation of its association with molecular subtype in a larger *HER2*+ cohort with gene expression data will be required. In addition, many of our data sets were highly heterogeneous, with images collected at a number of institutional sites and with a variety of scanners. Although the multi-institutional validation of our approach in cohorts with both high variability and homogeneous acquisition protocols (PRC2) is a promising sign regarding its robustness, further investigation into the sensitivity of peritumoral and intratumoral radiogenomic features to DCE-MRI acquisition is required.

Furthermore, biopsies provide only a small sample of tumor for comparison against imaging features that were computed and summarized across a large tumor volume. Thus, the histomorphometric associations reported in this work should be considered preliminary and will require more extensive correlation of radiologic, molecular, and pathologic data. Ultimately, although this signature’s association with *HER2*-E tumors will require further validation and will not replace PAM50 gene testing soon, our findings suggest the significant potential of quantitative radiomic analysis to characterize *HER2*+ biological characteristics pertinent to therapeutic response.

## Conclusions

In this study, a radiogenomic signature from the tumor and tumor environment characterizing the response-associated *HER2*-E subtype was identified, applied to estimate response to anti-*HER2* therapy, and then correlated with pathologic immune response on corresponding biopsy images. Future work will focus on validation of this signature, as well as its role in the outcome estimation and underlying biological basis, within a large, multi-institutional data set. With additional validation, these features could eventually result in a noninvasive method for helping to characterize tumor biological characteristics in *HER2*+ tumors and evaluate benefits of targeted therapy.

## Notes

Supplement. **eMethods.** Details on Design **eFigure 1.** Patient Selection Flowchart for the Molecular Subtype Discovery Cohort **eTable 1.** Comparison of Clinical Variables in Molecular Subtyping Discovery Cohort With the Original BrUOG 211B and TCGA-BRCA Study Populations **eFigure 2.** Overview of Radiogenomic Signature Development and Evaluation **eFigure 3.** ROC Curves for the Intra- and Peri-Tumoral Radiomics Model in Response Prediction Cohorts PR1 and PR2 **eFigure 4.** Training and Performance of Model for Lymphocyte Detection From H&E Slide Images of Pre-Treatment Biopsy Samples **eTable 2.** Lists of Top Features for Individual Intra- and Peri-Tumoral Regions and Combined Region Feature Sets, Along With Corresponding AUC in Identifying HER2-E in the Discovery Cohort and Identifying pCR in PR1 **eTable 3.** Repeated Feature Selection Experiments for HER2+ Molecular Subtyping Across All Intra- and Peri-Tumoral Regions Using Alternative, Non-Parametric Feature Pruning Approaches **eTable 4.** Risk stratification Table Comparing Intra-Tumoral Only and Combined Intra- and Peri-Tumoral Radiomics Model Within the Molecular Subtype Cohort **eTable 5.** Univariate and Multivariate Significance of Radiomic Classifier and Clinical Variables Within the Discovery Cohort (n = 42) **eTable 6.** Univariate and Multivariate Significance of Radiomic Classifier and Clinical Variables for the Prediction of pCR Within PR1 (n = 28) **eTable 7.** Radiologist Assessment of Fibroglandular Tissue (FGT) and Background Parenchymal Enhancement (BPE) for Validation Cohort PR1 According to BI-RADS Atlas 5th Edition **eFigure 5.** Correlation of HER2-E-Associated Feature Sets With Lymphocyte Density Within Tumor and Peripheral Tissue on Pre-Treatment Biopsy by Peri-Tumoral Distance **eReferences.**
