# Supplementary material for: Association of Peritumoral Radiomics With Tumor Biology and Pathologic Response to Preoperative Targeted Therapy for HER2 (ERBB2)–Positive Breast Cancer
Source: JAMA Netw Open. 2019 Apr 19;2(4):e192561. doi: 10.1001/jamanetworkopen.2019.2561 (PMC6481453; doi:10.1001/jamanetworkopen.2019.2561)
Supplement: Supplement. — eMethods. Details on Design eFigure 1. Patient Selection Flowchart for the Molecular Subtype Discovery Cohort eTable 1. Comparison of Clinical Variables in Molecular Subtyping Discovery Cohort With the Original BrUOG 211B and TCGA-BRCA Study Populations eFigure 2. Overview of Radiogenomic Signature Development and Evaluation eFigure 3. ROC Curves for the Intra- and Peri-Tumoral Radiomics Model in Response Prediction Cohorts PR1 and PR2 eFigure 4. Training and Performance of Model for Lymphocyte Detection From H&E Slide Images of Pre-Treatment Biopsy Samples eTable 2. Lists of Top Features for Individual Intra- and Peri-Tumoral Regions and Combined Region Feature Sets, Along With Corresponding AUC in Identifying HER2-E in the Discovery Cohort and Identifying pCR in PR1 eTable 3. Repeated Feature Selection Experiments for HER2+ Molecular Subtyping Across All Intra- and Peri-Tumoral Regions Using Alternative, Non-Parametric Feature Pruning Approaches eTable 4. Risk stratification Table Comparing Intra-Tumoral Only and Combined Intra- and Peri-Tumoral Radiomics Model Within the Molecular Subtype Cohort eTable 5. Univariate and Multivariate Significance of Radiomic Classifier and Clinical Variables Within the Discovery Cohort (n = 42) eTable 6. Univariate and Multivariate Significance of Radiomic Classifier and Clinical Variables for the Prediction of pCR Within PR1 (n = 28) eTable 7. Radiologist Assessment of Fibroglandular Tissue (FGT) and Background Parenchymal Enhancement (BPE) for Validation Cohort PR1 According to BI-RADS Atlas 5th Edition eFigure 5. Correlation of HER2-E-Associated Feature Sets With Lymphocyte Density Within Tumor and Peripheral Tissue on Pre-Treatment Biopsy by Peri-Tumoral Distance eReferences. [file jamanetwopen-2-e192561-s001.pdf]

Supplementary Online Content

Braman N, Prasanna P, Whitney J, et al. Association of peritumoral radiomics with tumor biology and pathologic response to preoperative targeted therapy for *HER2 (ERBB2)*–positive breast cancer. *JAMA Netw Open*. 2019;2(4):e192561. doi:10.1001/jamanetworkopen.2019.2561

eMethods. Details on Design

eFigure 1. Patient Selection Flowchart for the Molecular Subtype Discovery Cohort

eTable 1. Comparison of Clinical Variables in Molecular Subtyping Discovery Cohort With the Original BrUOG 211B and TCGA-BRCA Study Populations

eFigure 2. Overview of Radiogenomic Signature Development and Evaluation

eFigure 3. ROC Curves for the Intra- and Peri-Tumoral Radiomics Model in Response Prediction Cohorts PR1 and PR2

eFigure 4. Training and Performance of Model for Lymphocyte Detection From H&E Slide Images of Pre-Treatment Biopsy Samples

eTable 2. Lists of Top Features for Individual Intra- and Peri-Tumoral Regions and Combined Region Feature Sets, Along With Corresponding AUC in Identifying HER2-E in the Discovery Cohort and Identifying pCR in PR1

eTable 3. Repeated Feature Selection Experiments for HER2+ Molecular Subtyping Across All Intra- and Peri-Tumoral Regions Using Alternative, Non-Parametric Feature Pruning Approaches

eTable 4. Risk stratification Table Comparing Intra-Tumoral Only and Combined Intra- and Peri-Tumoral Radiomics Model Within the Molecular Subtype Cohort

eTable 5. Univariate and Multivariate Significance of Radiomic Classifier and Clinical Variables Within the Discovery Cohort (n=42)

eTable 6. Univariate and Multivariate Significance of Radiomic Classifier and Clinical Variables for the Prediction of pCR Within PR1 (n=28)

eTable 7. Radiologist Assessment of Fibroglandular Tissue (FGT) and Background Parenchymal Enhancement (BPE) for Validation Cohort PR1 According to BI-RADS Atlas 5th Edition

eFigure 5. Correlation of HER2-E-Associated Feature Sets With Lymphocyte Density Within Tumor and Peripheral Tissue on Pre-Treatment Biopsy by Peri-Tumoral Distance

eReferences.

This supplementary material has been provided by the authors to give readers additional information about their work.

© 2019 Braman N et al. *JAMA Network Open.*

eMethods. Details on Design

Annotation Protocol

Lesions were annotated by 7 readers, 6 of whom are board certified radiologists with 6-29 years contributing to radiology (DP, BNB, PT, ME, DDBB, KG) and 1 of whom is a medical doctor with extensive experience in medical imaging (KB). All readers with fewer than 5 years as a practicing radiologist (ME, DDBB, KG, KB) performed annotations in consensus with a senior radiologist (DP, 25 years practicing; BNB, 11 years practicing; PL, 7 years practicing). Lesions were annotated on three adjacent slices of DCE-MRI scans chosen to maximize tumor size while avoiding biopsy markers and artifacts. Radiologists were instructed to annotate the outer boundaries of tumor enhancement, including spiculation and internal non-enhancing regions surrounded by tumor (e.g. necrotic core).

Additional MRI acquisition Details for the Discovery Cohort

One or more 3D fat-saturated T1-weighted images were collected in the axial plane following contrast agent injection using a 1.5T (n=37) or 3.0T magnet (n=5). Pixel dimensions in the axial plane ranged from .50 mm x .50 mm to 1.06 x 1.06 mm, with an average of .70 mm x .70 mm+/- .14 mm. Slice thickness was on average 1.93 mm +/- .40 mm (range: 1-3 mm). Patients were injected with an average of 15.5 mL (range: 8-36 mL) of gadolinium-based contrast agent (Magnevist, Multihance, Gadavist, Optimark, or Prohance). Contrast agent dose was unavailable for five patients and contrast agent brand was unavailable for two.

Expanded Radiomic Descriptor Information

Laws. (1) 25 2-D Laws filters are derived by computing the outer product of combinations of the following 1-D filter vectors designed to capture specific textural patterns within an image. Each filter vector spans five pixels and is denoted as “P5,” where P is a letter representing the textural pattern captured by the filter.

1. Level (L5) – detects smoothness of intensity values.
    a. L5 = [1 4 6 4 1]
2. Edge (E5) – detects edges between regions with abrupt changes in intensity.
    a. E5 = [-1 -2 0 2 1]
3. Spot (S5) – detects speckled enhancement patterns.
    a. S5 = [-1 0 2 0 -1]
4. Wave (W5) – detects regularly oscillating local intensity patterns.
    a. W5 = [-1 2 0 -2 1]
5. Ripple (R5) – detects oscillating intensity patterns centered at region of extreme intensity.
    a. R5 = [1 -4 6 -4 1]

To obtain a feature vector, each filter is convolved with the image and the absolute value of filter response within all voxels contained within a region of interest are

© 2019 Braman N et al. *JAMA Network Open.*

concatenated. Features are named by the combination of filters applied in the y and x axes, e.g. L5E5 is the product of a level detection filter in the y axis and an edge detection filter in the x axis.

Gabor. (2) 2-D Gabor filters are computed by modulating a Gaussian kernel function with one of 48 sinusoidal plane waves. Each sinusoidal plane wave corresponds to a unique combination of one of six spatial wavelengths (2 pixels, 4 pixels, 8 pixels, 12 pixels, 16 pixels, 32 pixels, 64 pixels) and one of eight orientations (0°, 22.5°, 45°, 67.5°, 90°, 112.5°, 135°, 157.5°). Each Gabor filter is then convolved with the original image and values corresponding to filter response within the region of interest are concatenated.

Haralick GLCM. (3) Intensity values within the image are discretized into 64 bins from 0 to 63. Gray level co-occurrence matrices were computed within a sliding window of 5 pixels by 5 pixels. Intensity values outside the region of interest were ignored when computing GLCM statistics. The following GLCM descriptors were computed, as described in (3): entropy, energy, inertia, inverse difference moment, correlation, information measure of correlation 1, information measure of correlation 2, sum average, sum variance, sum entropy, difference average, difference variance, and difference entropy. GLCM statistics were concatenated within regions-of-interest, yielding 13 descriptor vectors per region.

Co-occurrence of local anisotropy gradients (CoLlAGe). (4) An image's intensity gradients in the x and y direction, F_x and F_y, are computed. Within a sliding 5 pixel square window, the dominant intensity gradient orientation (between 0°-360°) is computed via principal component analysis, resulting in a 2D array of equal size with the dominant gradient orientation value centered at the corresponding pixel of the original image. Metrics of the co-occurrence matrix are then applied to this gradient orientation image in the same manner as described above for Haralick GLCM features. The resulting 13 CoLlAGe descriptors are then the same 13 co-occurrence metrics (entropy, energy, inertia, inverse difference moment, correlation, information measure of correlation 1, information measure of correlation 2, sum average, sum variance, sum entropy, difference average, difference variance, and difference entropy) computed from the gradient orientation image, indicating the homogeneity of intensity gradient directionality within an image.

Permutation testing for ROC curve significance

A permutation testing framework was chosen to determine AUC significance due to its applicability in both a cross-validation and testing setting. For each individual model, permutation testing (5,6) was performed with Monte Carlo sample to assess whether model performance offered significant improvement over a random model. Each simulation consisted of 50,000 iterations.

- Testing: The locked down HER2-E classifier was applied to the validation cohort to obtain predicted probability of response. A test-statistic for the ROC curve was

© 2019 Braman N et al. *JAMA Network Open.*

computed using posterior probability from the classifier and ground truth response labels. Next, across 50,000 iterations, the ground truth pCR labels were randomly permuted. A second test statistic was computed from the original posterior probabilities and the randomly permuted labels. The proportion of runs where the test statistic corresponding to the randomly permuted ROC curve was greater than the true test statistic yields the p-value (5). 95% confidence intervals are twice the standard deviation in both directions from the mean AUC using non-permuted labels, assuming a normal distribution.

- Cross-Validation: for each iteration, DLDA classifiers were trained on both the original data and a data set with permuted class labels in a three-fold cross-validation setting. Posterior probabilities corresponding to classifiers trained using both the original and permuted labels were compiled for all patients across the three validation folds. Test statistics were computed for the ROC curve of each classifier. The p-value is the proportion of runs where the test statistic of the permuted classifier was greater than a classifier trained and tested using the ground truth (5). 95% confidence intervals are computed using the empirical probability distribution of the test statistic across all permutations and its variance (6).

Automated Lymphocyte Detection Model

A previously developed automated deep learning-based nuclei and lymphocyte detection model (7) was adapted and utilized to quantify the number of tumor infiltrating lymphocytes and tumor nuclei contained on digitized H&E tissue samples corresponding to biopsies obtained from the tumor periphery. Watershed nuclei segmentation (8) was compared with manual lymphocyte segmentation in 10 regions of interest (ROI) by a subspecialty-trained breast pathologist of 10+ years' experience. A Random Forest classifier (9) was trained to separate nuclei and lymphocyte segmentations based on shape, textural, and color features.

© 2019 Braman N et al. *JAMA Network Open.*

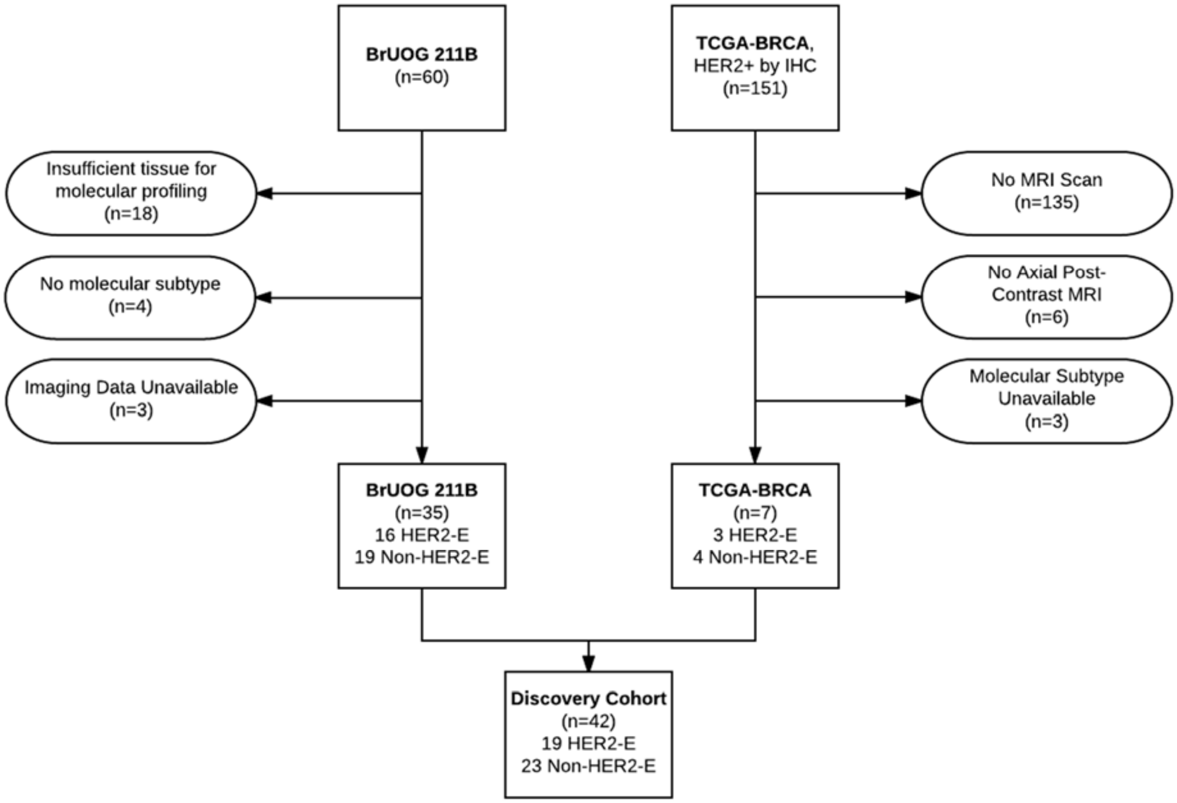

eFigure 1. Patient selection flowchart for the molecular subtype discovery cohort.

© 2019 Braman N et al. *JAMA Network Open.*

eTable 1. Comparison of clinical variables in molecular subtyping discovery cohort with the original BrUOG 211B and TCGA-BRCA study populations

	BrUOG 211B		HER2+ TCGA-BRCA		Discovery Cohort	
Variable	Original	Included	Original	Included		p-value
**Number of patients (No.)**	60	35	114	7	42	
**Age,** mean (SD), years[a]	52.0 (9.1)	51.7 (9.8)	58.4 (13.3)	54.0 (9.7)	51.3 (9.0)	p=.25[a]
**Receptor status**[b], No.						
ER+	30	18	79	7	25	P=.04[b]
PR+	20	14	62	5	19	P=.03[b]
**Stage**[b], No.						
I	0	0	7	4	4	P=.21[b,c]
II	39	21	72	3	24	
III	21	13	30	0	13	
IV	0	0	1	0	0	
NA	1	0	4	0	1	
**Molecular Subtype**, No.						P=.18 [b,d]
HER2-Enriched	16	16	39	3	19	
Luminal	19	15	34	4	19	
Basal	6	4	2	0	4	
N/A	19	0	39	0	0	

Abbreviations: ER, estrogen receptor; PR, progesterone receptor.

*Assessed by one-way analysis of variance (ANOVA) test between discovery cohort and original BrUOG 211B and TCGA-BRCA populations.
[b] Assessed by Pearson's chi-squared test between discovery cohort and original BrUOG 211B and TCGA-BRCA populations.
[c] Comparing distribution of Stage I, Stage II, and Stage III/IV
[d] Comparing only distribution of HER2-Enriched, Luminal, and Basal

© 2019 Braman N et al. *JAMA Network Open.*

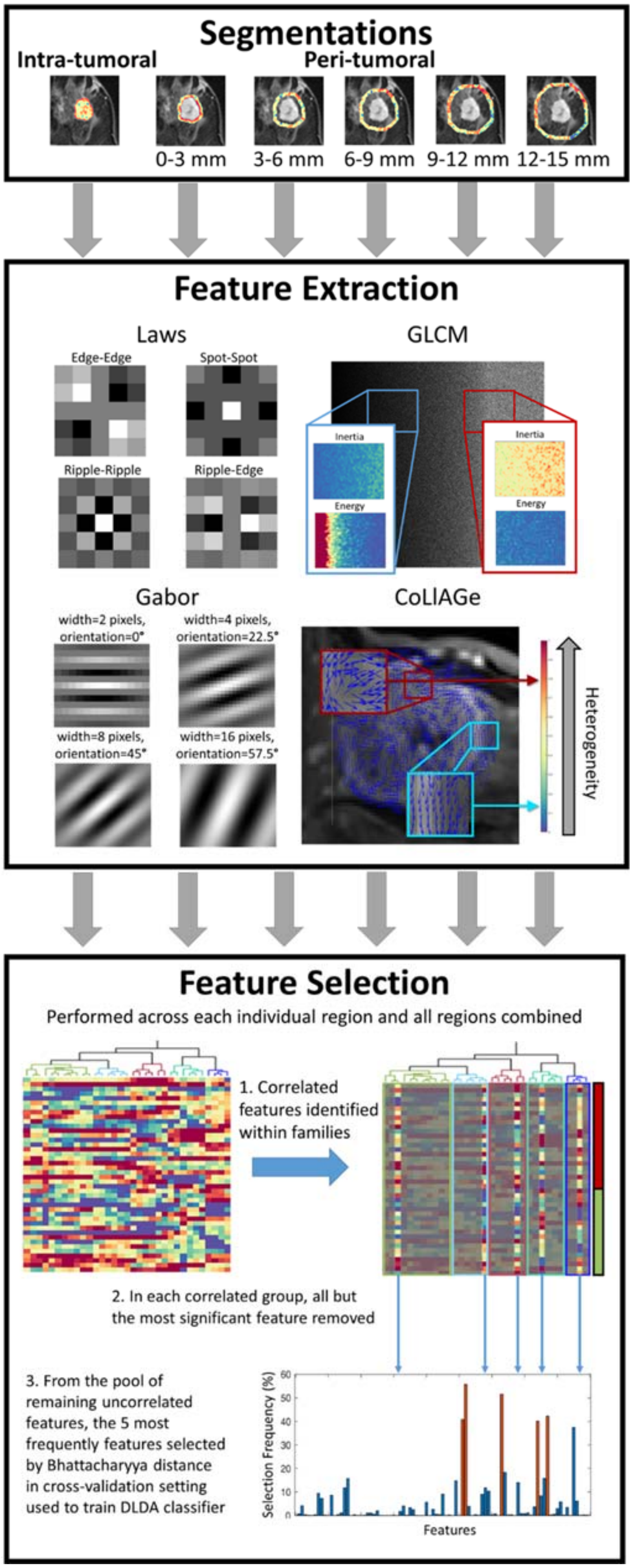

eFigure 2. Overview of radiogenomic signature development and evaluation. Tumor boundaries were manually annotated and used to derive five annular peri-

© 2019 Braman N et al. *JAMA Network Open.*

tumoral regions of interest of 3 mm each (top). Within each region, four families of 2D radiomic texture descriptors were computed and summarized via first order statistics (middle): Laws, Gabor, Gray Level Co-Occurrence Matrix (GLCM), and Co-occurrence of Local Anisotropy Gradients (CoLIAGe). A multi-stage feature selection approach was employed to select in cross-validation a set of 5 features associated with receptor status or molecular subtype. Diagonal linear discriminant analysis (DLDA) classifiers were trained and evaluated for each imaging signature in a cross-validation setting. Additionally, the HER2-E classifier was assessed for association with response in cohorts PR1 and PR2.

© 2019 Braman N et al. *JAMA Network Open.*

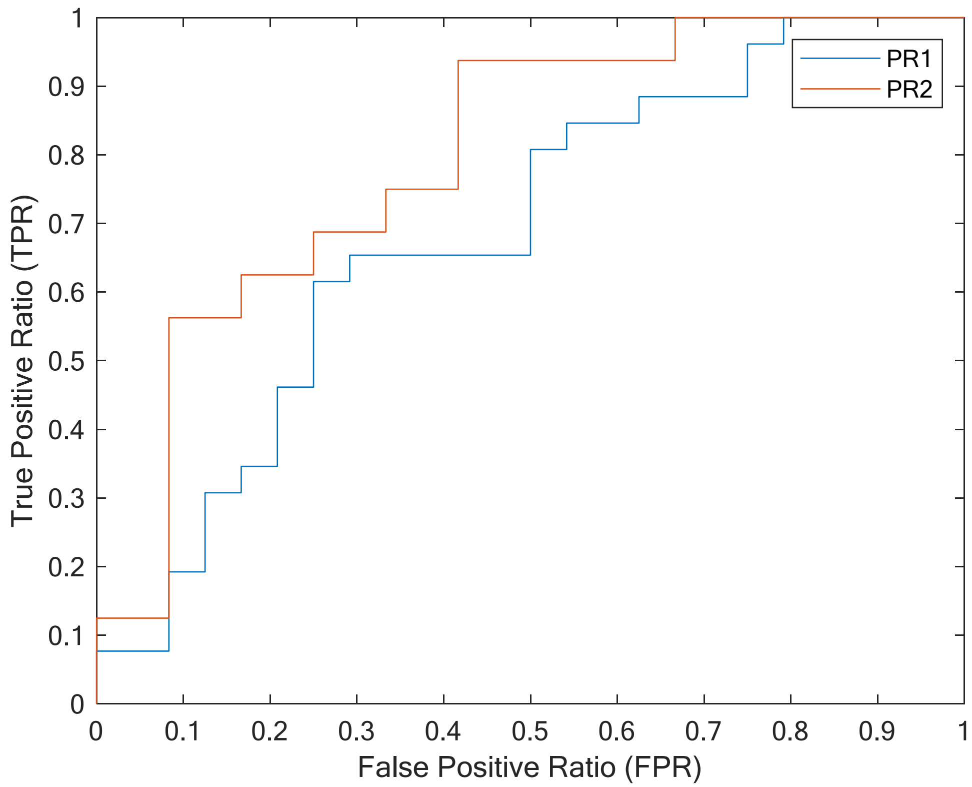

eFigure 3. ROC curves for the intra- and peri-tumoral radiomics model in response prediction cohorts PR1 and PR2.

© 2019 Braman N et al. *JAMA Network Open.*

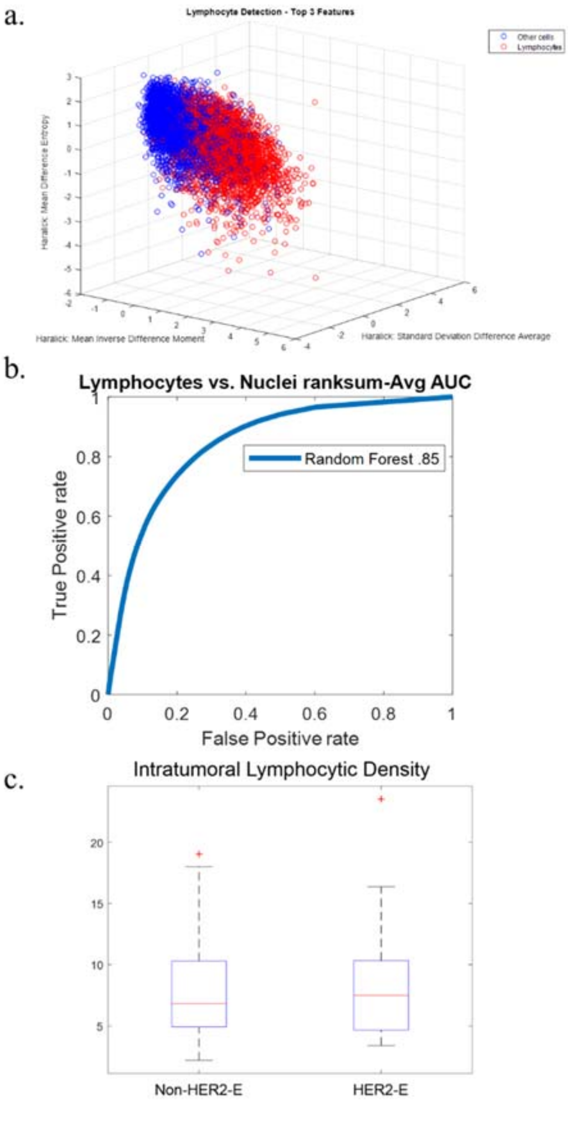

eFigure 4: Training and performance of model for lymphocyte detection from H&E slide images of pre-treatment biopsy samples. a) Top lymphocyte detection model features. Wilcoxon rank-sum feature selection was performed to identify the 44 features that best distinguished the nuclei of lymphocytes from those of other cells. The top three most discriminating features, all Haralick measures of nucleus texture, visually separate

© 2019 Braman N et al. *JAMA Network Open.*

lymphocytes (red) from other cells (blue). The full model incorporated 19 Haralick features, 22 shape features, and 3 color ratio features. b) Receiver Operating Characteristic curve for lymphocyte detection. A Random Forest classifier was trained in a 3-fold cross validation setting using the top 44 features. The classifier distinguished lymphocytic from other nuclei with an average AUC of .85. c) Lymphocytic density by subtype group. The trained classifier was applied to lymphocyte detection across whole H&E slide images. We observed a slight, but insignificant (p>.05), elevation in mean intra-tumoral lymphocytic density for HER2-E patients, consistent with previous findings on biopsy of the pre-treatment and a hand-counting of TIL density on the BrUOG dataset (10).

© 2019 Braman N et al. *JAMA Network Open.*

eTable 2: Lists of top features for individual intra- and peri-tumoral regions and combined region feature sets, along with corresponding AUC in identifying HER2-E in the discovery cohort and identifying pCR in PR1. p-values indicate significance of ROC curve AUC in permutation testing with random sampling.

	**Feature**			**Molecular Subtype**	**pCR**
**Region**	Group	Descriptor	Statistic	Discovery, AUC [95% CI]	PR1, AUC [95% CI]
Tumor					
	Gabor	w=4 px, θ=135°	Kurtosis	0.76*	0.66
	Laws	R5R5	Kurtosis	[0.69-0.84]	[0.43-0.88]
	Gabor	w=16 px, θ=112.5°	Kurtosis		
	Gabor	w=16 px, θ=45°	Kurtosis		
	CoLIAGe	Energy	Kurtosis		
0-3mm					
	CoLIAGe	Difference Average	Kurtosis	0.77*	0.44
	Gabor	w=8 px, θ=45°	Mean	[0.70-0.84]	[0.18-0.70]
	Gabor	w=16 px, θ=67.5°	Kurtosis		
	Gabor	w=8 px, θ=90°	Std Dev.		
	Gabor	w=8 px, θ=67.5°	Kurtosis		
3-6mm					
	Laws	R5R5	Kurtosis	0.83*	0.65
	CoLIAGe	Correlation	Mean	[0.76-0.90]	[0.39-0.91]
	Gabor	w=8 px, θ=67.5°	Skewness		
	Gabor	w=8 px, θ=45°	Median		
	CoLIAGe	Inertia	Kurtosis		
6-9mm					
	CoLIAGe	Difference Average	Skewness	0.83*	0.56
	CoLIAGe	Correlation	Mean	[0.77-0.88]	[0.32-0.81]
	Laws	R5S5	Kurtosis		
	CoLIAGe	Energy	Kurtosis		
	CoLIAGe	IDM	Kurtosis		
9-12mm					
	Gabor	w=16 px, θ=0°	Kurtosis	0.85*	0.53
	CoLIAGe	Inertia	Median	[0.79-0.90]	[0.28-0.78]
	Gabor	w=8 px, θ=22.5°	Kurtosis		
	Gabor	w=8 px, θ=112.5°	Kurtosis		
	Laws	R5W5	Skewness		
12-15mm					
	Laws	R5R5	Kurtosis	0.80*	0.63
	CoLIAGe	Difference Average	Median	[0.74-0.85]	[0.40-0.86]
	Gabor	w=8 px, θ=45°	Kurtosis		
	CoLIAGe	Correlation	Skewness		
	Gabor	w=32 px, θ=67.5°	Std Dev.		
COMBINED					
Tumor	Laws	R5R5	Kurtosis	0.89*	0.80*
Tumor	Gabor	w=16 px, θ=112.5°	Kurtosis	[0.84-0.93]	[0.61-0.98]
6-9mm	CoLIAGe	Energy	Kurtosis		
Tumor	Gabor	w=4 px, θ=135°	Kurtosis		
9-12mm	CoLIAGe	Inertia	Median		

*p<.005

Abbreviations: ER, estrogen receptor; PR, progesterone receptor.

© 2019 Braman N et al. *JAMA Network Open.*

AUC, area under the receiver operating characteristic curve; GLCM, Gray level co-occurrence matrix features. CoLIAGe, co-occurrence of local anisotropic gradient orientation features; px, pixels; w, width; θ, orientation.

© 2019 Braman N et al. *JAMA Network Open.*

eTable 3. Repeated feature selection experiments for HER2+ molecular subtyping across all intra- and peri-tumoral regions using alternative, non-parametric feature pruning approaches. Feature selection was repeated across the combined intra-tumoral and peri-tumoral feature pool using the following feature pruning approaches: (a) eliminating features by Spearman correlation > .6 and (b) eliminating features using an Elastic Net penalized regression with equal contributions by L1 and L2 regularization (11,12). Features listed in red were not identified in a given location in any feature discovery experiment. Features listed in orange were not included in the original combined intra- + peri-tumoral top feature set, but were identified as top features within individual annular regions. Repeating classification within the discovery cohort resulted in similar AUCs to feature pruning by Pearson correlation. Regardless of feature pruning approach, AUC remained strongly significant (p<.001).

	**Feature**			**PAM50**
**Region**	Group	Descriptor	Statistic	(Discovery, AUC)
**Original Top Features (Feature pruning by pearson correlation)**				
Tumor	Laws	R5R5	Kurtosis	.89 +/- .02
Tumor	Gabor	w = 16 px, θ = 112.5°	Kurtosis	
6-9 mm	CoLIAGe	Energy	Kurtosis	
Tumor	Gabor	w = 4 px, θ = 135°	Kurtosis	
9-12 mm	CoLIAGe	Inertia	Median	
**Feature pruning by Spearman correlation**				
Tumor	Laws	R5R5	Kurtosis	.84 +/- .02
Tumor	Gabor	w = 16 px, θ = 112.5°	Kurtosis	
6-9 mm	CoLIAGe	Energy	Kurtosis	
9-12 mm	CoLIAGe	Inertia	Median	
Tumor	Gabor	w = 16 px, θ = 45°	Kurtosis	
**Feature pruning by Elastic Net**				
Tumor	CoLIAGe	Entropy	Kurtosis	.87 +/- .03
6-9 mm	CoLIAGe	Energy	Skewness	
Tumor	Gabor	w = 16 px, θ = 112.5°	Kurtosis	
Tumor	Gabor	w = 16 px, θ = 45°	Kurtosis	
12-15 mm	CoLIAGe	Correlation	Skewness	

Abbreviations: ER, estrogen receptor; PR, progesterone receptor.
AUC, area under the receiver operating characteristic curve; GLCM, Gray level co-occurrence matrix features. CoLIAGe, co-occurrence of local anisotropic gradient orientation features; px, pixels; w, width; θ, orientation.

© 2019 Braman N et al. *JAMA Network Open.*

eTable 4. Risk stratification table comparing intra-tumoral only and combined intra- and peri-tumoral radiomics model within the molecular subtype cohort. In a cross-validation setting, HER2-E status was predicted using classifier with and without peri-tumoral features. Median classification results and 95% confidence intervals are displayed. Concordant predictions are between the two models are charted along the diagonal, while patients reclassified from Non-HER2-E to HER2-E or HER2-E to Non-HER2-E with the addition of peri-tumoral features are recorded in the top right and bottom left, respectively. Incorporating peri-tumoral features resulted in the reclassification of many patients to the correct ground truth category.

**Intra-tumoral radiomics model**		**Combined intra- and peri-tumoral radiomics model**		
		Non-HER2-E	HER2-E	**Total**
Non-HER2-E	N	17 [13.7 - 20.3]	6 [2.4 - 9.6]	23 [18.9 - 27.1]
	HER2-E	3 [1.3 - 4.7]	4 [0.7 - 7.3]	7 [3.9 - 10.1]
	Non-HER2-E	14 [11.4 - 16.6]	2 [0.6 - 3.4]	16 [13.3 - 18.7]
	Proportion HER2-E (%)	17.7 [9.1 - 26.4]	71.4 [46.9 - 96.0]	30.4 [20.4 - 40.4]
HER2-E	N	5 [2.3 - 7.7]	14 [10.5 - 18.4]	19 [14.9 - 23.1]
	HER2-E	0 [0 - 1.1]	12 [8.5 - 15.5]	12 [8.9 - 15.1]
	Non-HER2-E	5 [2.5 - 7.5]	2 [0.1 - 3.9]	7 [4.3 - 9.7]
	Proportion HER2-E (%)	0 [0 - 20.38]	85.7 [73.2 - 98.2]	63.2 [52.2 - 74.2]
**Total**	N	22 [19.3 - 24.6]	20 [17.9 - 23.1]	42
	HER2-E	3 [0.9 - 5.1]	16 [13.8 - 18.2]	19
	Non-HER2-E	19 [17.3 - 20.7]	4 [2.2 - 5.8]	23
	Proportion HER2-E (%)	13.6 [4.9 - 21.2]	80.0 [73.9 - 88.0]	45.2

© 2019 Braman N et al. *JAMA Network Open.*

eTable 5. Univariate and multivariate significance of radiomic classifier and clinical variables within the discovery cohort (n=42). Individually significant variables were included in a multinomial logistic regression model.

Variable	Univariate (p-value)	Multivariate (p-value)
Radiomics	<.001[a]	.007
Age	.78[a]	--
ER Status[c]	<.001[b]	.27
PR Status[c]	<.001[b]	.19
Stage	.58[b]	--

Abbreviations: ER, estrogen receptor; PR, progesterone receptor.

[a] Assessed by unpaired two-sided student's t-test.

[b] Assessed by Pearson's chi-squared test.

[d] Evaluated within n=41, as one patient did not have IHC-identified receptor status.

© 2019 Braman N et al. *JAMA Network Open.*

eTable 6. Univariate and multivariate significance of radiomic classifier and clinical variables for the prediction of pCR within PR1 (n=28). Since no clinical variables were found to be individually significant, all were included in multivariate analysis.

Variable	Univariate (p-value)	Multivariate (p-value)
Radiomics	.006 [a]	.03
Age	.93 [a]	.31
Size	.44 [a]	.53
ER Status	.38 [b]	.64
PR Status	.23 [b]	.41
Stage	.15 [b]	.10

Abbreviations: ER, estrogen receptor; PR, progesterone receptor;
[a] Assessed by unpaired two-sided student's t-test.
[b] Assessed by Pearson's chi-squared test.

© 2019 Braman N et al. *JAMA Network Open.*

eTable 7. Radiologist assessment of fibroglandular tissue (FGT) and background parenchymal enhancement (BPE) for validation cohort PR1 according to BI-RADS atlas 5th edition. Neither FGT (p=.52) nor BPE (p=.15) differed significantly between pCR and non-pCR when evaluated by two sample chi-squared test.

	**PR1**	
	**pCR**	**Non-pCR**
**Fibroglandular tissue**		
Fatty	1	0
Scattered	2	3
Heterogeneous	7	3
Extremely dense	6	6
**Background Parenchymal Enhancement**		
Minimal	1	3
Mild	5	6
Moderate	7	1
Marked	3	2

Abbreviations: pCR, pathological complete response; PR1, pathologic response cohort 1.

© 2019 Braman N et al. *JAMA Network Open.*

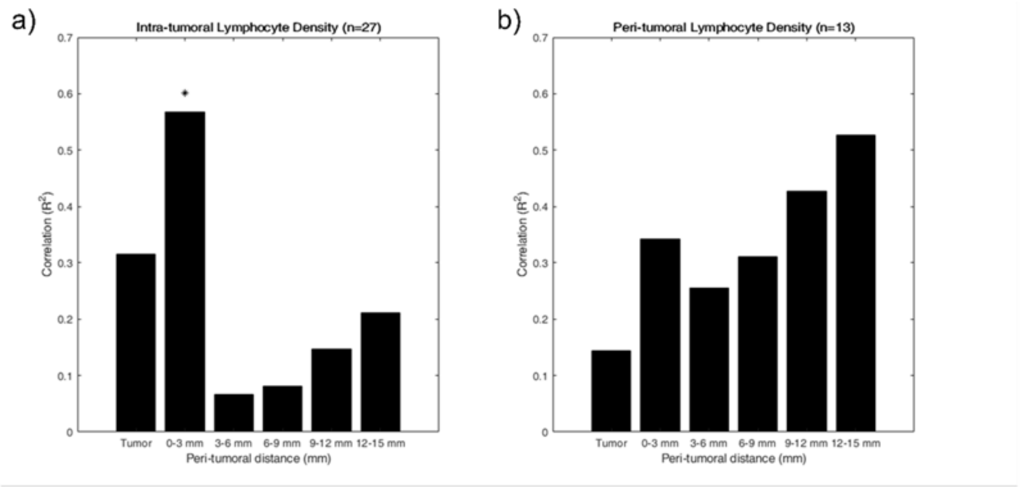

eFigure 5. Correlation of HER2-E-associated feature sets with lymphocyte density within tumor and peripheral tissue on pre-treatment biopsy by peri-tumoral distance. a) HER2-E-associated features 0-3mm from the tumor are significantly associated with TIL density (n=27). b) For patients whose biopsy contained peri-tumoral tissue (n=13), correlation between radiomic features and peripheral lymphocytic density was stronger with distance from the tumor, but was not significant.

© 2019 Braman N et al. *JAMA Network Open.*

eReferences.

1. Laws KI. Rapid Texture Identification. 1980 [cited 2016 Jun 17]. page 376–81. Available from: http://dx.doi.org/10.1117/12.959169

2. Fogel I, Sagi D. Gabor filters as texture discriminator. Biol Cybern. 1989;61:103–13.

3. Haralick RM, Shanmugam K, Dinstein I. Textural Features for Image Classification. IEEE Trans Syst Man Cybern. 1973;SMC-3:610–21.

4. Prasanna P, Tiwari P, Madabhushi A. Co-occurrence of Local Anisotropic Gradient Orientations (CoLlAGe): A new radiomics descriptor. Sci Rep. 2016;6:37241.

5. Neubert K, Brunner E. A studentized permutation test for the non-parametric Behrens–Fisher problem. Comput Stat Data Anal. 2007;51:5192–204.

6. Pauly M, Asendorf T, Konietschke F. Permutation-based inference for the AUC: A unified approach for continuous and discontinuous data. Biom J. 2016;58:1319–37.

7. Basavanhally AN, Ganesan S, Agner S, Monaco JP, Feldman MD, Tomaszewski JE, et al. Computerized Image-Based Detection and Grading of Lymphocytic Infiltration in HER2+ Breast Cancer Histopathology. IEEE Trans Biomed Eng. 2010;57:642–53.

8. Veta M, Diest PJ van, Kornegoor R, Huisman A, Viergever MA, Pluim JPW. Automatic Nuclei Segmentation in H&E Stained Breast Cancer Histopathology Images. PLOS ONE. 2013;8:e70221.

9. Ho TK. Random decision forests. Proc 3rd Int Conf Doc Anal Recognit. 1995. page 278–82 vol.1.

10. Varadan V, Gilmore H, Miskimen KLS, Tuck D, Parsai S, Awadallah A, et al. Immune Signatures Following Single Dose Trastuzumab Predict Pathologic Response to PreoperativeTrastuzumab and Chemotherapy in HER2-Positive Early Breast Cancer. Clin Cancer Res Off J Am Assoc Cancer Res. 2016;22:3249–59.

11. Regularization and variable selection via the elastic net - Zou - 2005 - Journal of the Royal Statistical Society: Series B (Statistical Methodology) - Wiley Online Library [Internet]. [cited 2018 May 8]. Available from: https://rss.onlinelibrary.wiley.com/doi/abs/10.1111/j.1467-9868.2005.00503.x

12. Friedman J, Hastie T, Tibshirani R. Regularization Paths for Generalized Linear Models via Coordinate Descent. J Stat Softw. 2010;33:1–22.

© 2019 Braman N et al. *JAMA Network Open.*

© 2019 Braman N et al. *JAMA Network Open.*
